# Improving Hox Protein Classification across the Major Model Organisms

**DOI:** 10.1371/journal.pone.0010820

**Published:** 2010-05-25

**Authors:** Stefanie D. Hueber, Georg F. Weiller, Michael A. Djordjevic, Tancred Frickey

**Affiliations:** Genomic Interactions Group, Research School of Biology, College of Medicine, Biology and Environment, The Australian National University, Canberra, Australian Capital Territory, Australia; American Museum of Natural History, United States of America

## Abstract

The family of Hox-proteins has been a major focus of research for over 30 years. Hox-proteins are crucial to the correct development of bilateral organisms, however, some uncertainty remains as to which Hox-proteins are functionally equivalent across different species. Initial classification of Hox-proteins was based on phylogenetic analysis of the 60 amino acid homeodomain. This approach was successful in classifying Hox-proteins with differing homeodomains, but the relationships of Hox-proteins with nearly identical homeodomains, yet distinct biological functions, could not be resolved. Correspondingly, these ‘problematic’ proteins were classified into one large unresolved group. Other classifications used the relative location of the Hox-protein coding genes on the chromosome (synteny) to further resolve this group. Although widely used, this synteny-based classification is inconsistent with experimental evidence from functional equivalence studies. These inconsistencies led us to re-examine and derive a new classification for the Hox-protein family using all Hox-protein sequences available in the GenBank non-redundant protein database (NCBI-nr). We compare the use of the homeodomain, the homeodomain with conserved flanking regions (the YPWM and linker region), and full length Hox-protein sequences as a basis for classification of Hox-proteins. In contrast to previous attempts, our approach is able to resolve the relationships for the ‘problematic’ as well as ABD-B-like Hox-proteins. We highlight differences to previous classifications and clarify the relationships of Hox-proteins across the five major model organisms, *Caenorhabditis elegans*, *Drosophila melanogaste*r, *Branchiostoma floridae*, *Mus musculus* and *Danio rerio*. Comparative and functional analysis of Hox-proteins, two fields crucial to understanding the development of bilateral organisms, have been hampered by difficulties in predicting functionally equivalent Hox-proteins across species. Our classification scheme offers a higher-resolution classification that is in accordance with phylogenetic as well as experimental data and, thereby, provides a novel basis for experiments, such as comparative and functional analyses of Hox-proteins.

## Introduction

One of the most exciting puzzles in developmental research is posed by the highly conserved set of Hox-protein transcription factors and how they set up specific body patterns along the anterior-posterior axis of bilateral animals. Mis-expression of Hox-genes can lead to drastic phenotypes, such as the famous four-winged fly [1] or a fly sprouting legs from its head where antennae should be [2]. In humans, Hox-gene mis-expression can result in the formation of extra vertebrae, digits or genital malformations [3], [4]. Another striking peculiarity of Hox-proteins is that the corresponding genes are clustered on the chromosome and expressed along the anterior-posterior axis of the organism in a manner consistent with the relative positions of the genes on the chromosome [5], [6]. Similar Hox-gene clusters have been found in all bilateral organisms examined to date. Research on Hox-proteins is preferentially conducted in model organisms such as *Caenorhabditis elegans* (nematode), *Drosophila melanogaster* (fruit fly), *Mus musculus* (mouse) or *Danio rerio* (zebrafish) since these organisms are easy to manipulate genetically and tools are available that circumvent the lethality of many Hox-gene mis-expressions.

The extent to which information about the molecular function of a Hox-protein gained from one model organism is transferable to other organisms can be assessed by comparing the presumed functionally equivalent proteins from different species. If, for example, over-expression of a Drosophila Hox-protein and the presumed functionally equivalent protein from mouse exhibit a similar phenotype in Drosophila, we can have higher confidence that this phenotype is due to a conserved feature in the proteins we compare. Insights gained from experiments analyzing a Hox-protein feature responsible for such a phenotype will therefore most likely be transferable to other species, including humans. Identification of presumed functionally equivalent proteins is usually performed by inferring a sequence-based evolutionary history for the proteins, the underlying assumption being that the amino acid sequence of a protein reflects its ancestry and function. Although Hox-proteins are critical to the correct development of bilateral organisms, the identification of functionally equivalent Hox-proteins in the different model organisms is not always straight forward.

All Hox-proteins contain a highly conserved 60 amino acid sequence motif, the homeodomain [7], [8]. The high degree of sequence conservation led to this domain being used as the main feature in determining how the various Hox-protein encoding genes are related to one another [9], [10]. The homeodomains of Hox-proteins were generally found to exhibit greater sequence similarity to the homeodomains of proteins encoded by genes in comparable positions in the Hox-clusters of other species than to adjacent genes in the Hox-cluster of the same species. It was therefore proposed that a ‘prototypic’ or ‘ancestral’ Hox-cluster had evolved from a single Hox-gene via tandem duplication and subsequent divergence [1], [11] and that the common ancestor of all bilateral organisms must have contained a partially differentiated, ‘prototypic’ Hox-cluster containing approximately six genes [11], [12]. This ‘prototypic’ cluster is thought to have further diverged and in some cases multiplied by whole genome duplications [13], to give rise to the different types and numbers of Hox-clusters present in our model organisms of interest. These include a single, fairly dispersed cluster in the nematode *C*. *elegans* (6 Hox-genes) [14], a single interrupted Hox-cluster in the fruit fly *D*. *melanogaster* (8 Hox-genes) [10], a single Hox-cluster in the prechordate amphioxus *B*. *floridae* (14 Hox-genes) [15], four clusters in the mouse *M*. *musculus* as well as humans *Homo sapiens sapiens* (39 Hox-genes each) [16] and seven clusters in the zebrafish *D*. *rerio* (48 Hox-genes) [17].

**Figure 1:**
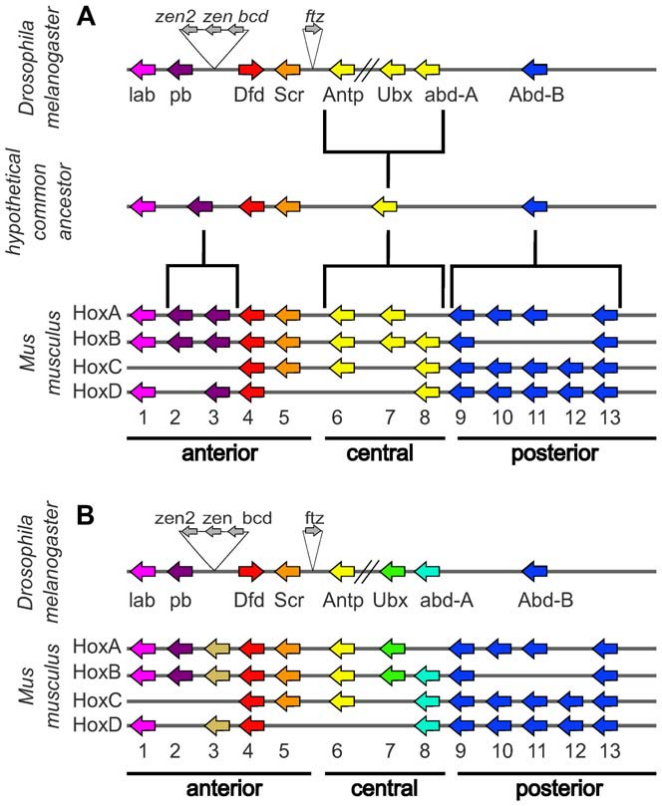
Classification schemes for *Drosophila melanogaster* and *Mus musculus* Hox-proteins. The Hox-protein coding genes are depicted and classified according to the encoded proteins. A) Phylogeny-based classification of Hox-proteins according to their inferred ancestry based on their similarities across the homeodomains. Such classifications often include representation of a hypothesized common ancestor. B) Frequently depicted Hox-classification scheme in which synteny was used to further resolve the ANTP, UBX, ABD-A vs. Hox6, Hox7, Hox8 grouping. The difference between these classification schemes is best exemplified by the classification of the Drosophila ANTP, UBX and ABD-A proteins in relation to the vertebrate Hox6, Hox7 and Hox8 groups of proteins. In A) these proteins are grouped together and it remains unclear which of the proteins in this group are to be regarded as functionally most similar across the species. In B) these proteins are grouped according to the relative positions of their genes within the Hox-cluster.

Some Hox-proteins with clearly distinct functions and distinct sets of downstream genes [18]-[20] proved difficult to classify due to their nearly identical homeodomains. This is best exemplified by the classification of the Drosophila ANTP, UBX and ABD-A proteins in relation to the vertebrate Hox6, Hox7, and Hox8 protein groups. Due to their high sequence similarity, these proteins are believed to have arisen from the same gene in the ‘ancestral’ cluster [21]. There are two distinct ways these proteins have previously been classified (Figure 1). A) Phylogeny-based classification schemes infer an evolutionary history for the Hox-proteins based on their similarities across the homeodomains. The exact evolutionary relationships of many Hox-proteins can be reliably determined, however, some groups of proteins with (inferred) common ancestry cannot be reliably fully resolved. Proteins within such unresolved groups are often classified as one further unresolvable group of homologs/orthologs/co-orthologs. A summary of phylogeny-based classification schemes is depicted in Figure 1A [11], [12], [21]–[26]. A disadvantage of these classifications is that it remains unclear which of the proteins within unresolved groups are to be regarded as most functionally similar across species. B) Synteny-based schemes, a second prominent classification method, attempt to resolve this issue by further subdividing unresolved groups using the relative positioning of the genes within the Hox-cluster. Examples thereof can be seen in [22], [27]–[32] (summarized in Figure 1B). This latter classification scheme relies on the assumption that the position of the genes in the Hox-cluster reflects ancestry or function in the organism. Clear examples where this is not the case are provided by a number of arthropod species and the sea urchin, in which inversions seem to have changed the relative order of the Hox-genes in the Hox-cluster [23], [33]. Another example where synteny-based classification may not be appropriate is the ‘problematic’ set of central Hox-proteins, i.e. Drosophila ANTP-UBX-ABD-A in relation to vertebrate Hox6-Hox7-Hox8. Consistent with the phylogeny-based classification scheme (Figure 1A), we can hypothesize that Drosophila and vertebrates independently triplicated an ‘ancestral’ ANTP-UBX-ABD-A/Hox6-Hox7-Hox8 protein. This would lead to a Hox-cluster with the exact same gene order and sequence similarities we observe, but a synteny-based assignment would wrongly predict co-orthologous proteins to be orthologous. While this may seem trivial, it is not. Co-orthologous proteins are more likely to have diverged considerably in their function than truly orthologous proteins as, due to their independent duplication, they are also expected to be subject to independent selection pressures. Such a mis-classification of co-orthologous proteins as orthologous could lead researchers to compare, across different model organisms, the downstream effects of proteins that have different functions.

**Figure 2:**
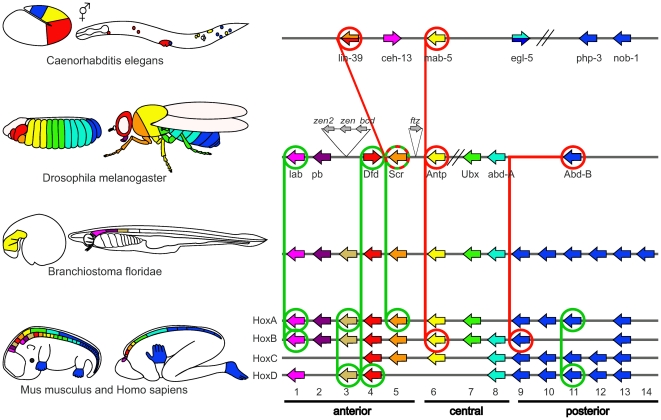
Experiments supporting and conflicting with assignments of presumed functionally equivalent Hox-proteins. The left side depicts the known expression patterns for the Hox-genes in four model organisms. The right side illustrates the corresponding chromosomal organization of the Hox-genes. Individual Hox-genes are represented by colored arrows. The color code is the same as used in the classification scheme in Figure1B (synteny-based). In *C. elegans* some genes have two colors, as a single equivalent protein in the other model organisms could not be determined. Experiments analyzing functional equivalence are shown as lines connecting genes in different Hox-clusters. Green and red lines indicate, respectively, supporting and conflicting experimental evidence regarding presumed functional equivalence of the proteins. The diagram is not to scale for either organism size, gene size or cluster size. Comparison of the Drosophila LAB and vertebrate HOXB1 was carried out in *D*. *melanogaster* using a *Gallus gallus* (chicken) HOXB1.

Fortunately, it is possible to assess the accuracy of Hox-protein classification schemes by examining whether the Hox-proteins, expected to be functionally similar based on the classification, actually lead to similar mis-expression phenotypes *in vivo*. The results of functional comparison studies are summarized in Figure 2. Both the phylogeny- and synteny-based classification schemes are in agreement with the experimental evidence for: Drosophila Labial (LAB) vs. chicken (*Gallus gallus*) HOXB1 (rescue experiment) [34], Drosophila Deformed (DFD) vs. human HOXD4 [35], Drosophila Sex combs reduced (SCR) vs. murine HOXA5 [36] (both ectopic expression phenotype comparisons) as well as various paralogs within mouse [37]–[39]. However, studies of this type have been unable to confirm functional equivalence across species for any of the central or posterior Hox-proteins [40]–[42] (Figure 2).

One specific example in which the experimental evidence does not support the synteny-based classification scheme that predicts ANTP to be equivalent to Hox6, UBX to Hox7 and ABD-A to Hox8, is provided by a comparative analysis of ectopic expression phenotypes in Drosophila for the Drosophila Antennapedia (ANTP) and murine HOXB6 proteins. For this example, it is important to know that most Hox-proteins are capable of inducing antenna to generic leg phenotypes in Drosophila [43]. HOXB6 is able to induce partial transformation of antennae into generic legs, however, it is not able to induce the specific leg type (T2) induced by ectopic expression of Drosophila ANTP [40]. As such, HOXB6 does not appear to be a better functional equivalent to ANTP than other Hox-proteins. A further example where previous classifications are questionable is provided by ectopic expression of Drosophila ABD-B and murine HOXB9 in Drosophila [41]. While some phenotypes were in common between ABD-B and HOXB9, e.g. the ability to induce ectopic abdominal-type denticles in addition to thoracic ones, most of the HOXB9 phenotypes were clearly distinct from those induced by ABD-B. In embryos, for example, HOXB9 expression was unable to induce ectopic posterior spiracles or create ABD-B-like morphologies of denticles and sensory organs. HOXB9 also exhibited additional functions usually attributed to other Hox-proteins, but not ABD-B, such as partial transformation of the posterior head into the dorsal thorax.

Knowing which proteins provide the best functional equivalents across different species is pivotal to predicting and understanding Hox-protein function such as, for example, differentiating between the ‘co-selective binding’ (specific DNA binding) and ‘widespread binding’ (transcriptional activity regulation once bound to the DNA) models defined by Biggin and McGinnis [44]. The two most prevalent classification schemes for Hox-proteins (Figure 1) coincide and agree with experimental evidence in their classification of the anterior Hox-proteins. However, the classification of the central and posterior Hox-proteins is less clear. For the above experiments, ANTP vs. HOXB6 and ABD-B vs. HOXB9, the schemes either provide insufficient resolution (phylogeny) or predict proteins with differing functions to be functionally equivalent (synteny). In either case the classification schemes do not provide any estimates to which extent the function of the predicted equivalent proteins will be comparable. Furthermore, the relationship beween the posterior amphioxus Hox-proteins (Hox9 to Hox15) to the corresponding vertebrate proteins (paralogy groups Hox9 to Hox13) is unclear and needs to be resolved [45].

In an attempt to improve upon previous classifications, we examined all Hox-protein sequences available in the GenBank non-redundant protein database (NCBI-nr). Our aim was to improve three aspects of the existing classification schemes: I) correct potential mis-classifications of Hox-proteins, II) refine the classification for the insufficiently resolved groups of Hox-proteins and III) provide estimates as to how comparable the most similar Hox-proteins from different organisms are likely to be. We present a pairwise sequence similarity based classification of the family of Hox-proteins with special emphasis on the major model organisms. To help resolve the relationship of the ‘problematic’ central group of Hox-proteins we define an extended Hox-homeodomain encompassing their YPWM motif, linker region and homeodomain. The classification scheme we provide is in complete accordance with the published experimental evidence and provides a more detailed classification of the ANTP, UBX, ABD-A and Hox6, Hox7, Hox8 as well as the ABD-B, vertebrate Hox9-13 (vHox) and amphioxus Hox9–15 (AmphiHox) groups of proteins than previous classification schemes. The results indicate the utility of including the YPWM motif and linker region for the classification of Hox-proteins and strongly suggest that these elements have a role in determining Hox-protein function. The detailed classification of these groups provides novel and experimentally testable predictions for functionally comparable pairs of Hox-proteins across the major model organisms.

## Results

**Figure 3:**
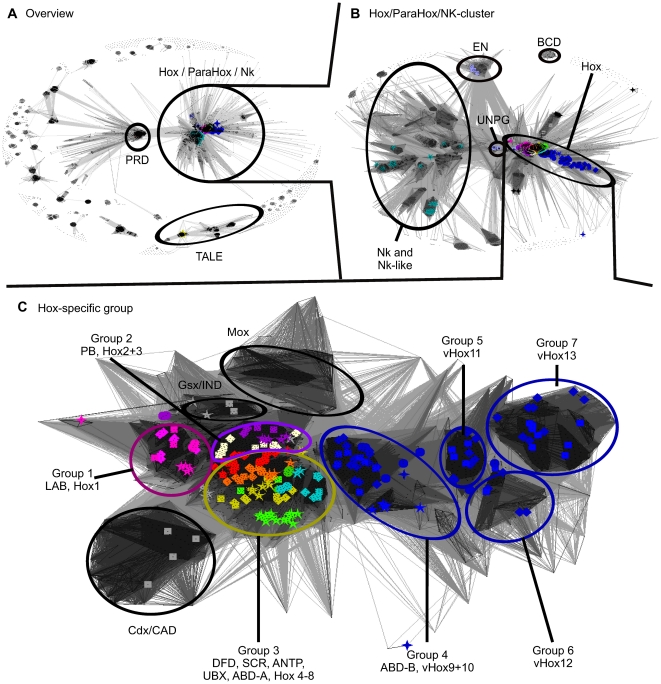
Homeodomain-only clustering. A) CLANS overview of the pairwise sequence similarities for the set of 15,788 sequences identified as potentially Hox-related. P-cutoff  =  10^−15^; coloring: red  =  Paired, yellow  =  Irx, turquoise  =  NK-cluster. B) Detailed view of the Hox/ParaHox/Nk-cluster identified in A). P-cutoff  = 10^−18^; coloring as in A). C) Detailed view of the Hox and Hox-like sequences identified in B) (including the non-Hox-protein ‘Cdx/Cad’, Gsx/Ind and Mox clusters). P-cutoff  = 10^−18^; coloring as in Figure 1B. 5-pointed stars represent *Drosophila melanogaster*, 4-pointed stars represent *Caenorhabditis elegans*, circles represent *Branchiostoma floridae*, rectangles represent *Mus musculus* and rhomboids represent *Danio rerio* sequences.

Using PSI-BLAST, we identified a set of 15,788 sequences with potential relevance to the classification of Hox-proteins. A CLANS analysis of this set (Figure 3A) revealed that, in addition to the sequences for all known Hox-proteins, we also retrieved the protein sequences for related, but clearly distinct protein families such as the NK-cluster, ParaHox, Iroquois (TALE) and paired. The paired/paired-like and TALE class proteins are generally regarded as distantly related sister groups to the Hox-proteins, while Nk and ParaHox sequences are generally regarded as more closely related sister groups. Our set of 15,788 sequences therefore contained sequence families related to Hox-proteins, yet clearly outside the scope of our analysis. This indicated we had exhaustively retrieved all Hox and Hox-like sequences relevant to our classification. As the Iroquois (TALE) and paired groups were of no further interest, we removed the corresponding sequence clusters as well as any clusters not connected to known Hox-protein sequences and focused solely on the main group containing all Hox/ParaHox and NK-cluster sequences (Figure 3B). Subsequent clustering allowed us to identify the various sub-groups present in this set and focus on the main group of interest (Hox and Hox-like) (Figure 3C).

**Figure 4 pone-0010820-g004:**
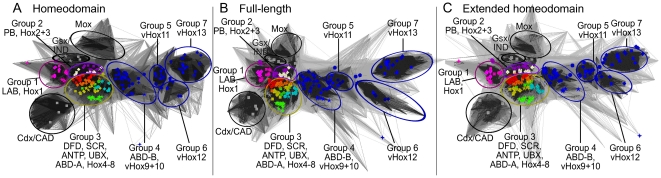
Overview of Hox-clusters generated by CLANS. A) 60 amino acid homeodomain sequences, B) Full-length protein sequences and C) Extended homeodomain sequences. Irrespective of the type of sequence used, the CLANS analyses generate very similar cluster maps in which the seven major groups identified in Figure 3C can be found in comparable positions.

We performed three types of cluster analyses using sequence similarities derived from: I) The 60-amino acid homeodomain identified by McGinnis et al. [7], II) Full-length Hox-protein sequences and III) Extended homeodomain sequences, as described in the methods section. As can be seen in Figure 4, all three clusterings produce very similar cluster-maps for the Hox-protein family. Some differences in the detailed grouping of sequences in these three sets are apparent. The differences are predominantly restricted to the central Hox-proteins and are discussed in the corresponding sections.

### I) 60 amino acid homeodomain clustering

Visual and automated analysis (‘network-clustering’) of the Hox and Hox-like sequences we identified in Figure 3B revealed 7 major Hox-protein sequence similarity clusters (Figure 3C). These 7 groups combine sequences from the various model organisms in accordance with previous classification schemes:

Group 1) Drosophila Labial (LAB), amphioxus and vertebrate Hox1 sequences.

Group 2) Drosophila Proboscipedia (PB) and Zerknüllt (ZEN), as well as amphioxus and vertebrate Hox2 and 3 sequences.

Group 3) Drosophila Deformed (DFD), Sex combs reduced (SCR), Antennapedia (ANTP), Ultrabithorax (UBX) and Abdominal-A (ABD-A), *C. elegans* LIN-39 and MAB-5, as well as the amphioxus and vertebrate Hox4-8 sequences.

Groups 4–7) Four distinct groups formed by ABD-B and ABD-B-like proteins, containing the Drosophila ABD-B, amphioxus Hox9-12, and vertebrate Hox9-13 sequences. The amphioxus Hox13–15 sequences do not cluster as part of groups 4–7.

Hox15, NOB-1 and EGL-5 are outliers to these groups and discussed separately.

### Groups 1 and 2

Our assignment of sequences to groups 1 and 2 is in agreement with both of the widely used classification schemes presented in Figure 1 as well as experimental data.

**Figure 5 pone-0010820-g005:**
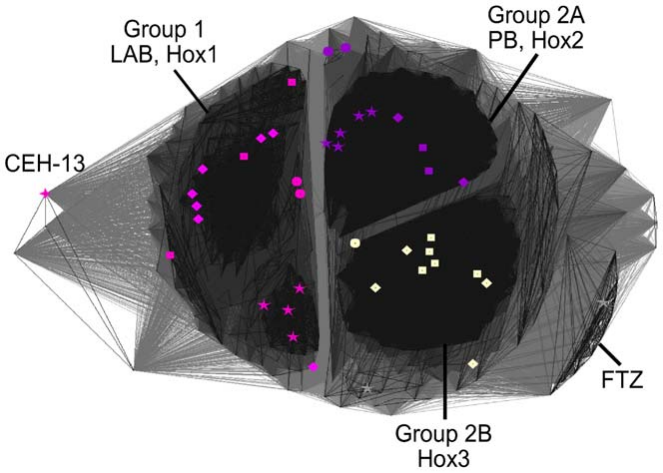
Detailed clustering of sequences from groups 1 and 2 identified in **Figure 3C**. P-cutoff  = 10^−18^. Coloring as in Figure 1B: pink  =  LAB+Hox1, purple  =  PB+Hox2, beige  =  Hox3. 5-pointed stars represent *Drosophila melanogaster*, 4-pointed stars represent *Caenorhabditis elegans*, circles represent *Branchiostoma floridae*, rectangles represent *Mus musculus* and rhomboids represent *Danio rerio* sequences. Group 2 sequences can further be subdivided into groups 2A (Hox2-like) and 2B (Hox3-like).

Our group 1 combines Drosophila Labial (LAB) and the Hox1 proteins. Chicken HOXB1 was previously shown to be able to rescue phenotypes caused by mutations in the Drosophila *lab* Hox-gene [34]. The only difference to previous classifications for the sequences in group 1 regards the assignment of *C. elegans* CEH-13. In our analysis, CEH-13 is most similar to group 1, but in contrast to previous classifications, is identified as an outlier to this group (see Figure 5).

Our group 2 encompasses Hox2- and Hox3-like sequences. Sequences from the Hox2 and Hox3 families are known to be highly similar and were previously postulated to have diverged from a common *Hox2*/*3* ‘ancestral’ gene [21]. Our analysis further subdivides sequences from group 2 into two distinct sub-groups 2A and 2B. Sub-group 2A contains Drosophila PB and vertebrate Hox2-like sequences, while sub-group 2B contains the vertebrate Hox3-like sequence (and no Drosophila sequences) (Figure 5).

### Group 3

**Figure 6 pone-0010820-g006:**
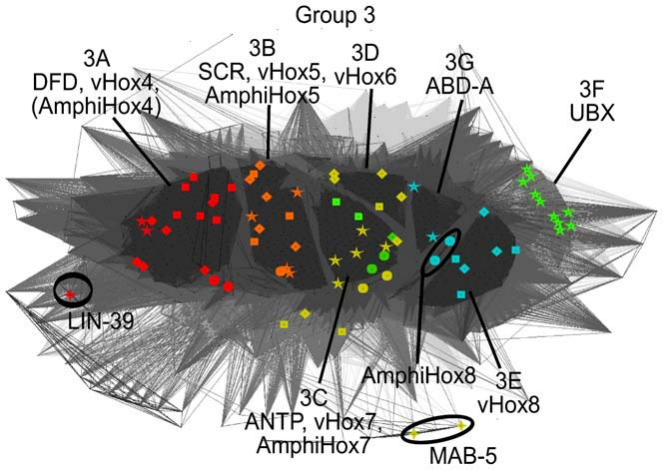
2D clustering of group 3. P-value 10^−25^; coloring as in Figure 1B: red  =  DFD+Hox4, orange  =  SCR+Hox5, yellow  =  ANTP+Hox6, green  =  UBX+Hox7, light-blue  =  ABD-A+Hox8. 5-pointed stars represent *Drosophila melanogaster*, 4-pointed stars represent *Caenorhabditis elegans*, circles represent *Branchiostoma floridae*, rectangles represent *Mus musculus* and rhomboids represent *Danio rerio* sequences. Separate clusters are formed by the protein sequences for 3A  =  DFD/Hox4, 3B  =  SCR/Hox5, 3C  =  ANTP/Hox7, 3D  =  Hox6, 3E  =  Hox8, 3F  =  UBX and 3G  =  ABD-A.

Our group 3 encompasses several Drosophila Hox-proteins known to exhibit different developmental functions [18], [19]. We further analyzed this group to gain a better view of its sub-structure. Detailed examination revealed seven distinct sequence similarity sub-clusters 3A-F (Figure 6).

Sub-cluster 3A encompasses the Drosophila DFD, amphioxus and vertebrate Hox4 proteins. This sub-cluster contains two sequences for which an interspecies functional comparison was previously carried out: mouse HOXD4 vs. Drosophila DFD. Ectopic expression of murine HOXD4 in *D. melanogaster* caused similar phenotypes and regulatory activities as ectopic expression of the Drosophila DFD protein, i.e. the ability to auto-induce the transcription of DFD [35]. In contrast to previous classifications, our analysis predicts *C. elegans* LIN-39, although grouping most closely to sub-group 3A, not to be part of this sub-group. In the literature, assignment of LIN-39 is ambiguous, as it is sometimes regarded as equivalent to DFD [46] and sometimes as equivalent to SCR [14]. Previous functional comparisons only analyzed the function of Drosophila SCR and ANTP in *C. elegans*, but not DFD. In ectopic expression experiments, Drosophila SCR was able to mimic LIN-39 effects on QL and QR migration, but the rescue of the *lin-39* mutant CP-neuron phenotype was limited [42]. Based on the cluster analysis of group 3, we predict that sequences from group 3A (DFD-like) should provide better replacements for LIN-39 than group 3B (SCR-like) sequences, however, we predict neither to provide a good functional replacement.

Sub-cluster 3B encompasses the Drosophila SCR protein as well as the amphioxus and vertebrate Hox5 proteins. SCR and mouse HOXA5 proteins were previously shown to be functionally equivalent in Drosophila ectopic expression experiments [36].

Sub-cluster 3C encompasses ANTP, amphioxus Hox7 and vertebrate Hox7 sequences. The amphioxus Hox6 and zebrafish Hoxb6b sequences cluster in proximity to, but not as part of this group.

Sub-cluster 3D encompasses mouse HOXC6, zebrafish HoxC6a and HoxC6b proteins.

Mouse HOXA6, HOXB6 and zebrafish HoxB6a proteins do not preferentially cluster with either sub-group 3C or 3D when pairwise similarity values derived from the 60 amino acid homeodomain are used.

Sub-cluster 3E encompasses the vertebrate Hox8 proteins, sub-cluster 3F the Drosophila UBX sequences and sub-cluster 3G the Drosophila ABD-A sequences.

The amphioxus Hox8 sequences cluster between groups 3C, 3D, 3E and 3G and cannot be preferentially assigned to any of the above.

The classification we derive from sub-clusters 3C-G differs significantly from the widely used classification schemes depicted in Figure 1. While the phylogeny-based classification scheme (Figure 1A) cannot further resolve the relationships between the Drosophila ANTP, UBX, ABD-A and vertebrate Hox6, Hox7, Hox8 proteins, the synteny-based classification scheme (Figure 1B) assigns ANTP as equivalent to Hox6, UBX to Hox7, and ABD-A to Hox8. The synteny-based classification scheme conflicts with the available experimental data [40] specifically for those proteins for which it differs from our classification. Our classification predicts Hox7 to be a better functional equivalent for ANTP than Hox6.

Peculiarities of this group are further discussed in sections II) Full-length clustering and III) Extended homeodomain clustering.

### Group 4

In contrast to previous classification schemes, which did not provide any resolution of the ABD-B like proteins, our classification separates these into four groups, 4–7.

**Figure 7 pone-0010820-g007:**
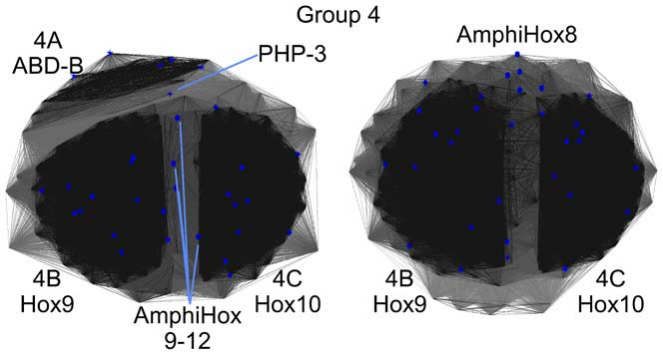
2D-representations of a 3D homeodomain clustering of group 4. This group was clustered in 3D, as a 2D clustering was unable to provide sufficient resolution to show the sub-structure present within this group. The two figures differ by a 90° rotation around the X-axis; P-value cutoff  = 10^−15^. Three major groups are visible: 4A  =  Drosophila ABD-B, 4B  =  vertebrate Hox9 and 4C  =  vertebrate Hox10 sequences.

Our group 4 combines sequences of *C. elegans* PHP-3, Drosophila ABD-B, amphioxus Hox9–12 and vertebrate Hox9 and Hox10, which form three separate sub-groups 4A–C (Figure 7). Sub-group 4A contains Drosophila ABD-B sequences. The *C. elegans* PHP-3 protein sequence clusters closely to this group and may, with caution, be regarded as part of this group. PHP-3 is the only *C. elegans* sequence that clusters with a Drosophila Hox-protein. Sub-group 4B contains the vertebrate Hox9 and sub-group 4C vertebrate Hox10 sequences. The amphioxus Hox9–12 sequences do not cluster with any of the three main sub-groups 4A–C. They do, however, cluster firmly in-between the vertebrate Hox9 (4B) and 10 (4C) sequence groups.

Previous experiments showed most of the phenotypes induced by murine HOXB9 to be clearly distinct from those induced by Drosophila ABD-B [41]. Consistent with these experimental data, the corresponding proteins appear in separate sub-clusters in our analysis (Figure 7). Our analysis indicates, that *C*. *elegans* PHP-3 should provide a better functional equivalent to Drosophila ABD-B than any of the vertebrate or amphioxus Hox-proteins.

### Groups 5–7

**Figure 8 pone-0010820-g008:**
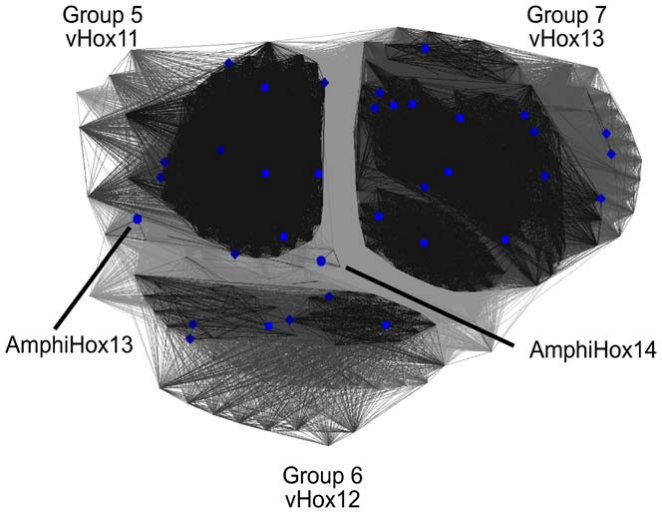
2D homeodomain clustering of groups 5 to 7. P-value cutoff  = 10^−18^. Group 5 combines vertebrate Hox11, group 6 vertebrate Hox12 and group 7 vertebrate Hox13 sequences. Amphioxus Hox13 and Hox14 sequences can be seen grouping in close proximity, but not as part of group 5 (vHox11).

Our groups 5, 6 and 7, shown in Figure 8, encompass the vertebrate Hox11 (group 5), vertebrate Hox12 (group 6) and vertebrate Hox13 (group 7) sequences. The amphioxus Hox13 and Hox14 sequences cannot be assigned to any one of the groups. The amphioxus Hox13 sequence clusters in-between groups 5 and 6 with a slight preference to group 5. Amphioxus Hox14 is most similar to amphioxus Hox13, but does not cluster with any of the above groups. Within this set of amphioxus and vertebrate Hox-proteins, the most comparable sequences between the model organisms are amphioxus Hox13 and vertebrate Hox11, although the sequences should not be expected to provide functionally equivalent proteins.

Functional equivalence studies for proteins within groups 5–7 have only been performed comparing paralogous Hox11 proteins in mouse (HOXA11 vs. HOXD11) [37] and therefore do not provide us with information regarding the functional equivalence of Hox-proteins from different species. However, these experiments are consistent with our classifications as the Hox11 proteins identified as functionally equivalent co-localize in the same cluster in our analysis.

### Hox15, NOB-1 and EGL-5

Two extreme outliers exist in the ABD-B-like group of proteins: amphioxus Hox15 and *C. elegans* NOB-1. The amphioxus Hox15 sequence (from *B. lanceolatum*, as no Hox15 was available for *B. floridae*) is highly divergent from the other ABD-B class proteins and, although it is most sequence similar to vertebrate Hox13-like proteins, it does not not cluster with, or in proximity to, any Hox sequence from any of the model organisms we examine. The *C. elegans* NOB-1 sequence has diverged considerably from other Hox-proteins and can only be classified as derived from an ABD-B-type sequence.

The *C*. *elegans* protein EGL-5 has previously been classified as either a Drosophila ANTP-like [47], ABD-A-like [27] or, most often, ABD-B-like Hox-protein [14]. However, based on our analysis, EGL-5 should not be assigned to any of the Hox-protein groups. The EGL-5 Hox-protein sequence is so different from other Hox-proteins that it lies outside the ‘Hox-specific’ cluster displayed in Figure 3C. This figure includes all other *C. elegans* Hox-proteins, all Drosophila and vertebrate Hox-proteins, as well as non-Hox-proteins of the ‘Cdx/CAD’, ‘Gsx/IND’ and ‘Mox’ class, and the non-Hox, but Hox-derived, FTZ and ZEN proteins. This indicates that EGL-5 differs in sequence from other Hox-proteins to a greater extent than some of the non-Hox-proteins present in Figure 3C, e.g. FTZ and ZEN that are regarded as having lost their Hox-protein function. EGL-5 should therefore, at best, be regarded as a highly derived member of the Hox-protein family that is unlikely to exhibit a function comparable to any of the Hox-proteins in our examined model organisms.

### II) Full-length clustering

**Figure 9:**
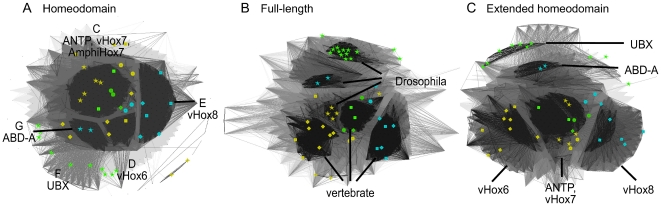
Comparative 2D clustering of the Drosophila ANTP, UBX, ABD-A and the vertebrate Hox6, Hox7 and Hox8 sequence groups. The sequence groups were identified based on Figure 6. Depicted are the clusters for homeodomain, full-length as well as extended homeodomain sequences. Coloring as in Figure 1B: yellow  =  ANTP+Hox6, green  =  UBX+Hox7, light-blue  =  ABD-A+Hox8. 5-pointed stars represent *Drosophila melanogaster*, circles represent *Branchiostoma floridae*, rectangles represent *Mus musculus* and rhomboids represent *Danio rerio* sequences. A) 60 amino acid homeodomain sequences. Separate clusters are apparent for ANTP+Hox7, ABD-A, UBX and Hox8 sequences. Vertebrate Hox6 sequences are dispersed across multiple clusters. B) Full-length protein sequences. Separate clusters are formed for arthropod and vertebrate sequences. ANTP lies in close proximity to Hox6 and Hox7 sequences. C) Extended homeodomain sequences. ANTP and Hox7 appear in one cluster while, ABD-A, UBX, Hox8 and vertebrate Hox6 sequences form separate groups. These figures were generated using the same CLANS P-value cut-off of 10^−26^.

Sequences outside the homeodomain are known to influence Hox-protein function, as exemplified by two ABD-B isoforms that carry out different functions in Drosophila and vary only in their sequence outside the homeodomain [48]. We therefore re-analyzed our cluster maps using sequence similarity values derived from the full-length and extended homeodomain sequences (see Methods), in particular for the ‘problematic’ Hox-protein assignments we identified based on the 60 amino acid homeodomains. While the general overview of the Hox-protein family as a whole was very similar between our three datasets (Figure 4), clustering of full-length sequences has a tendency to generate ‘species’ or ‘clade’-specific clusters compared to clustering of 60 amino acid homeodomain sequences (compare Figures 9A and 9B) without producing the types of groups we seek, i.e. groups combining both chordate and arthropod sequences.

### III) Extended homeodomain clustering

In an attempt to resolve this problem, we defined an extended homeodomain as ranging from the YPWM/W motif, N-terminal to the homeodomain, to the C-terminal end of the homeodomain itself. These additional regions have recently been shown to be relevant for Hox-protein function [49], [50]. Clustering the Hox-protein sequences based on their pairwise similarity across this extended homeodomain leads to a clustering very similar to the one generated by the 60 amino acid homeodomain, but allows us to resolve the ‘problematic’ ANTP-UBX-ABD-A/Hox6-Hox7-Hox8 group.

From the detailed view of group 3 sequences in Figure 9, it is apparent that Drosophila ANTP sequences cluster most closely to vertebrate Hox7 sequences. In addition, the vertebrate Hox6 sequences, which were difficult to assign to a group based on the homeodomain alone, now group consistently with one another and form a cluster well separated from the vertebrate Hox7, Hox8 or any of the Drosophila proteins (Figure 9C).

By combining the information gleaned from the cluster maps for I) 60 amino acid homeodomain clustering, II) Full-length clustering and III) Extended homeodomain clustering for the sequences in group 3 (Drosophila ANTP, UBX, ABD-A and vertebrate Hox6, Hox7, Hox8 sequences), we suggest the following:

- Using the 60 amino acid homeodomain, all sequences in group 3 are very similar to one another and the relationships between these sequences cannot be reliably further elucidated.
- Our clustering of the full-length sequences in group 3 does not help identify functionally equivalent proteins, as Drosophila and vertebrate sequences do not cluster together. Instead, three separate groups are generated for both the arthropod (ANTP, UBX, ABD-A) and vertebrate (vHox6, vHox7, vHox8) sequences. A higher sequence similarity between ANTP and vHox7 is apparent from the clustering (and to a lesser extent to vHox6), but it is not sufficient to warrant combining these vertebrate and arthropod-specific groups.
- Our clustering of the extended homeodomain sequences resolves their relationship. As in the 60 amino acid homeodomain clustering, ANTP and Hox7 sequences cluster together. However, in contrast to the above cluster maps, all of the vHox6 sequences form a clearly distinct and separate cluster. This map also shows the Drosophila UBX and ABD-A sequences form separate cluters most similar to the ANTP/Hox7 group and, similarly, the Hox6 and Hox8 sequences form separate clusters most similar to the ANTP/Hox7 group. This indicates that vHox6 should not be assigned as equivalent to ANTP, nor should vHox7 to UBX or vHox8 to ABD-A. vHox7 proteins are most sequence similar to ANTP and should be regarded as the most equivalent set within the ‘problematic’ Hox-proteins.

This is precisely the type of sequence similarity relationship one would expect to find for two groups of co-orthologous sequences. Based on this information we would predict ANTP and Hox7 to have retained the most ‘ancestral-type’ sequence and UBX, ABD-A, Hox6 and Hox8 sequences to have been independently duplicated and adapted to new functions in the Drosophila and vertebrate lineages.

It also should be noted that the amphioxus Hox6 and Hox8 sequences consistently cluster closely to the ANTP/Hox7 group of sequences, irrespective of whether the homeodomain or the extended homeodomain is used. This indicates that amphioxus is likely to have retained, in all three proteins, an ‘ancestral’-type of sequence and, possibly, function.

The *C. elegans* sequence MAB-5, generally classified as being orthologous to ANTP, could not be determined as preferentially clustering with any of these groups. As such, MAB-5 appears to be a highly derived version of a putative ‘ancestral’ ANTP/Hox7-like protein. This is also in accordance with previously published results, showing that ANTP, though a better functional substitute for MAB-5 than SCR, is still considerably different to MAB-5 in each of the functions analyzed in *C*. *elegans* [42]. That *C. elegans* contains a single, highly derived, ANTP/Hox7-like protein, and amphioxus contains three similar ANTP/Hox7-like proteins, further supports the hypothesis that the common ancestor of Drosophila and vertebrates may only have had a single ANTP/Hox7-like protein, rather than the fully differentiated set of three proteins present in both lineages today.

## Discussion

**Figure 10:**
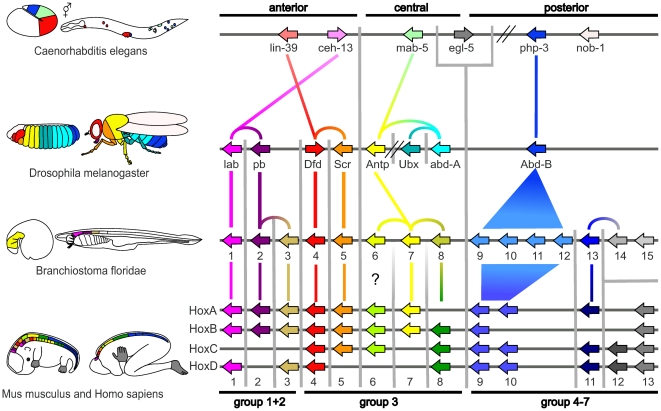
Proposed classification. The organisms are ordered to show the clearest representation of the Hox-proteins most similar in sequence and thus expected to be most similar in function. The figure is not supposed to indicate that Drosophila is descended from Caenorhabditis or that the chordates descended from Drosophila. Vertical gray lines delineate sequence similarity groups. Colored lines linking Hox-genes indicate which Hox-proteins are most sequence similar to one another. Links within a species indicate a presumed multiplication, or loss, of the corresponding proteins in a lineage, while links between species indicate the most sequence similar pairs of Hox-proteins in these species. The colors are used to represent groups of similar sequences, except for the ‘non-colors’ white and gray. These ‘non-colors’ indicate proteins with considerable sequence divergence to any other sequence in the model organisms we compare. Zebrafish is not depicted as the assignment between tetrapods and zebrafish is clear.

Comparative and functional analyses have been hampered by difficulties in predicting which proteins from different model organisms should be regarded as functionally equivalent. Due to the considerable difficulties in selecting which protein sequences to actually compare, the field has had difficulties identifying evolutionarily conserved or functionally relevant amino acid motifs in Hox-proteins [51]. The overview and higher-resolution classification of Hox-proteins we present will hopefully help clarify some of the existing controversial assignments and facilitate the selection of comparable sequences across species. We have summarized the main findings in Figure 10.

One of the advantages of our classification regards the assignment of the Hox-proteins ANTP, UBX and ABD-A in relation to the vertebrate/chordate Hox6, Hox7 and Hox8 proteins. As their homeodomains are very similar, the location of their genes on the chromosome has in the past been used to determine which proteins are to be regarded as functionally equivalent: with ANTP being assigned as equivalent to Hox6, UBX to Hox7 and ABD-A to Hox8. However, a functional comparison of HOXB6 and ANTP did not support this assignment [40]. Our cluster analysis indicates that ANTP, UBX and ABD-A, as well as the Hox6, Hox7 and Hox8 sequence groups are very similar in their sequences, but that the Hox7 group of proteins is likely to provide a better functional equivalent for ANTP than the Hox6 group. In addition, vertebrate Hox9–13 sequences appear to have diverged considerably from the *C. elegans* PHP-3 and Drosophila ABD-B proteins. The *C. elegans* PHP-3 protein may therefore prove to be a more suitable candidate for rescue and comparative experiments in Drosophila than the vertebrate Hox9 protein, which previously failed to mimic multiple Drosophila ABD-B functions in such experiments [41].

We also noticed that the current numbering scheme employed for the ABD-B class proteins does not accurately reflect the sequence similarities between amphioxus and vertebrates. The different numbers, sequence conservations and placement of ABD-B-like proteins in the cluster maps indicate that the set of amphioxus Hox9–12 proteins should only be expected to rescue functions that are shared between vertebrate Hox9 and 10 proteins and vice versa. Amphioxus Hox13 is most similar to vertebrate Hox11, though the sequences have noticeably diverged and a fully functionally equivalent protein should not be expected. The amphioxus Hox14 protein cannot be further classified beyond being most similar to amphioxus Hox13 and showing some similarity to the vertebrate Hox11–13 groups of proteins. Some ABD-B class proteins diverge in their sequence so significantly that they appear to be either specific to amphioxus, such as the amphioxus Hox15 protein, or specific to vertebrates, such as the vHox12 and vHox13 groups of proteins. These proteins are unlikely to have functionally comparable Hox-proteins in, respectively, either vertebrates or amphioxus. For the Abd-B type proteins, our analysis indicates that there is no vertebrate protein that can be expected to exhibit virtually identical functions to an amphioxus, *C*. *elegans* or Drosophila Hox-protein.

In summary, our classification highlights some key differences in its prediction of putative functionally equivalent Hox-proteins compared to currently used classification schemes. We identified questionable assignments in the synteny-based classification scheme that correspond to those proteins for which experimental studies revealed significant functional differences. Our clustering approach provides a novel, robust and purely sequence-based classification scheme that is in accordance with the available experimental data. The clusters allow us to identify which sequence similarity groups are present, but also provide a graphical representation of how similar the sequences in a group are to one another in relation to other Hox-proteins. This provides a more graduated classification and facilitates selecting the right proteins for future experiments. Hox-proteins classified within one group would be of interest for functional equivalence studies or to understand which amino acids influence the specific interaction of Hox-proteins with the DNA or other proteins. Hox-proteins with more divergent sequences might be of greater interest for evolutionary studies or when trying to find common, shared or even divergent amino acid motifs responsible for the differences in function.

The cluster maps provide an overview of the pairwise sequence similarities between Hox-proteins and allow graduated estimates for the expected similarity of the function of Hox-proteins. Our improved classification scheme (Figure 10) and the differences we highlight, specifically in respect to the widely used synteny based classification, provide a number of testable predictions regarding which Hox-proteins should be functionally most similar across the major model organisms.

## Methods

Our aim was to address inconsistencies between current Hox-protein classifications and experimental results by deriving a novel classification scheme using all sequences available in the NCBI-nr database (version May 18^th^ 2009) (ftp.ncbi.nlm.nih.gov/blast/db/FASTA/nr.gz). To identify all sequences relevant to this analysis, the *Drosophila melanogaster* Hox-proteins (LAB, PB, DFD, SCR, ANTP, UBX, ABD-A and ABD-B) (see Table 1 and Appendix S1 for the seed sequences used) were used to seed iterative PSI-BLAST (version 2.2.20) [52] searches (inclusion value -h 10^−5^). These searches were run to convergence or up to 50 iterations against the NCBI-nr database. From these searches, all high-scoring segment pairs (HSPs) with E-values better than 10 to any of the eight Drosophila seed sequences were gathered and the corresponding full-length sequences were extracted. This provided a non-redundant set of 15,788 sequences, that we used for subsequent analyses. The aim of this step was to exhaustively identify all Hox and Hox-related sequences (true-positives) present in the NCBI-nr database without missing any sequences relevant to our analysis (false negatives) while keeping the total number of sequences returned (true positives + false positives) within the limits of what subsequent analysis tools could handle.

**Table 1:** Seed sequence identifiers.

Protein name	Isoform	GenBank ID	NCBI-gi-number	RefSeq ID
LAB	lab-PA	AAF54098	GI:17136284	NP_476613.1
PB	pb-PA	AAF54089	GI:45549028	NP_476669.3
	pb-PB	AAS65120	GI:45553273	NP_996163.1
	pb-PC	AAS65119	GI:45553271	NP_996162.1
	pb-PD	AAS65118	GI:45553267	NP_996161.1
DFD	Dfd-PA	AAF54083	GI:17137270	NP_477201.1
SCR	Scr-PA	AAF54082	GI:24644694	NP_524248.2
	Scr-PB	AAS65103	GI:45553277	NP_996165.1
	Scr-PC	AAS65104	GI:45553275	NP_996164.1
ANTP	Antp-PD	AAS65109	GI:45553295	NP_996174.1
	Antp-PE	AAS65107	GI:45553291	NP_996172.1
	Antp-PF	AAS65108	GI:45553285	NP_996169.1
	Antp-PG	AAS65105	GI:45553293	NP_996173.1
	Antp-PH	AAG22205	GI:45553299	NP_996176.1
	Antp-PI	AAS65111	GI:45553283	NP_996168.1
	Antp-PJ	AAS65112	GI:45553281	NP_996167.1
	Antp-PK	AAS65106	GI:45553279	NP_996166.1
	Antp-PL	AAS65113	GI:45553287	NP_996170.1
	Antp-PM	AAS65114	GI:45553297	NP_996175.1
	Antp-PN	AAS65110	GI:45553289	NP_996171.1
UBX	Ubx-PA	AAF55355	GI:17985969	NP_536752.1
	Ubx-PB	AAF55356	GI:18079282	NP_536748.1
	Ubx-PC	AAN13719	GI:24647525	NP_732173.1
	Ubx-PD	AAN13717	GI:24647521	NP_732171.1
	Ubx-PE	AAN13718	GI:24647523	NP_732172.1
	Ubx-PF	AAS65158	GI:45553381	NP_996219.1
ABD-A	abd-A-PA	AAF55359	GI:17136422	NP_476693.1
	abd-A-PB	AAF55360	GI:24647534	NP_732176.1
	abd-A-PC	ACZ94928	GI:281361946	NP_001163632.1
ABD-B	Abd-B-PA	AAF55363	GI:24647542	NP_650577.1
	Abd-B-PB	AAF55362	GI:24647540	NP_524896.2
	Abd-B-PC	AAF55364	GI:24647544	NP_732180.1
	Abd-B-PD	AAN13723	GI:24647546	NP_732181.1
	Abd-B-PE	AAS65159	GI:45553383	NP_996220.1

This table provides a list of the sequences used to seed the PSI-BLAST searches. These consist of all available isoforms for the *Drosophila melanogaster* Hox-proteins (LAB, PB, DFD, SCR, ANTP, UBX, ABD-A and ABD-B). Provided are the protein names, the specific isoform used and the corresponding GenBank identifier, NCBI-gi-number and RefSeq-ID. The FASTA sequences are also available from the seed sequence file (Appendix S1) in the supplementary materials.

- Sequence similarity of the homeodomain: the 60 amino acid homeodomain sequence as defined by McGinnis et al. (1984) [7] was extracted for each of the eight *D*. *melanogaster* Hox-proteins above, aligned in AlnEdit [53] using Muscle [54] (see Appendix S2 for the homoedomain alignment), manually refined and a global hidden Markov model (HMM) was derived from this alignment using HMMer (version 2.3.2) [55]. The resulting HMM was calibrated with 5000 replicates and used to identify all putative homeodomains present in the above set of 15,788 full-length sequences. Regions matching the HMM with E-values better than 10 were extracted and analyzed using the pairwise sequence similarity visualization and analysis program CLANS [56], [57].
- Sequence similarity of full-length proteins: the above described set of 15,788 full-length sequences was analyzed using CLANS. Mis-annotated proteins and constructs in the NCBI-nr database were identified based on the number of homeodomains present in the protein sequences (Hox-proteins in the model organisms of interest contain no more than one homeodomain per protein) and discarded. Homeodomains were identified as described in I). Some mis-annotated or artificial construct sequences were present in our set of 15,788 full-length sequences. For example, the entry gi|194043948 (*Sus scrofa*) contains, as a single amino acid sequence, the protein sequences encoded by five Hox-genes of the mammalian HOXD cluster. This and any protein sequence containing more than one homeodomain was removed prior to the CLANS analysis.
- Sequence similarity of an extended homeodomain: as described by Joshi et al. [49], the YPWM motif N-terminal to the homeodomain is involved in modifying the ability of Hox-proteins to bind DNA through interaction with additional factors such as Extradenticle (Exd). As the sequence region between the YPWM motif and the homeodomain was shown to interact with the minor groove in the Hox/Exd interaction experiment, we also classified Hox-proteins using an extended homeodomain encompassing the YPWM motif, the homeodomain and the sequence region in-between (referred to as the ‘linker’ region). This extended homeodomain was extracted for each of the eight *D*. *melanogaster* Hox-proteins, aligned in AlnEdit using Muscle (see Appendix S3 for the extended homoedomain alignment) and a global HMM was derived from this alignment using HMMer. The resulting HMM was calibrated with 5000 replicates and used to identify the corresponding extended homeodomain regions in the 15,788 Hox-related full-length sequences. The sequence regions matching the HMM with E-values better than 10 were analyzed using CLANS.

Analysis of the three sequence regions described above (I homeodomain only, II full-length, III extended homeodomain) was performed using more stringent settings. By default, all CLANS analyses were performed using a P-value cut-off of 10^−15^. Clusters were detected via both the automated ‘network-clustering’ method and visual interpretation of the map (see Appendix S4 for details about the ‘network-clustering’ approach). Automated ‘network-clustering’ provides a quick method for cluster-detection. However, as conflicting information is obscured by automated clustering appoaches, but retained visually in the CLANS map, preference was always given to the visual interpretation. Subsequent analysis of the identified Hox-clusters using more stringent P-value cut-offs improved the resolution for some of the clusters. Where differing from the default, the P-value cut-off is stated in the figure legend and CLANS parameter files (supplementary materials). All sequences and parameters present in Figures 3, 4, 5, 6, 7, 8, 9 as well as those used for the Hox15, NOB-1 and EGL-5 protein comparisons (not shown) are made available as text files containing the FASTA-format sequences (Appendix S5) and the parameters (Appendix S6). The supplementary material section also includes a file (Appendix S7) linking to a website containing the CLANS program as well as the CLANS save-files generated in the course of this analysis. Creation of CLANS files for Figures 3A and B may require up to 12 GB of RAM, all other files can be viewed with 3 GB of RAM or less.

## Supporting Information

Appendix S1 Seed sequences. The FASTA-format sequences used to seed the PSI-BLAST searches (also see Table1).
(0.02 MB TXT)

Appendix S2 Homeodomain alignment. The multiple sequence alignment of homeodomains from which a Profile-Hidden-Markov-Model (HMM) was derived. This HMM was subsequently used to identify the homeodomains of the sequences in our set of interest.
(0.00 MB TXT)

Appendix S3 Extended homeodomain alignment. The multiple sequence alignment of extended homeodomains from which a Profile-Hidden-Markov-Model (HMM) was derived. This HMM was subsequently used to identify the extended homeodomains of the sequences in our set of interest.
(0.00 MB TXT)

Appendix S4 CLANS network-clustering. Overview of the “network-clustering” approach as implemented in CLANS. Aim of this approach is to automatically identify groups of sequences with greater similarity to each other than to the rest.
(0.24 MB PDF)

Appendix S5 CLANS sequences. A Zip archive containing the various groups of sequences used in our CLANS analyses. The archive provides one file with FASTA-format sequences for each of the similarity maps displayed in figures 3–9. In addition, the archive also contains the set of FASTA-format sequences used for our comparison of Hox15, NOB-1 and EGL-5.
(2.41 MB ZIP)

Appendix S6 CLANS parameters. A Zip archive containing text files specifying the parameters used for each of the generated CLANS cluster maps.
(0.01 MB ZIP)

Appendix S7 CLANS links to save-files. A short text file providing web-links to the CLANS program and the CLANS save-files for each of the similarity maps used in our analysis.
(0.00 MB TXT)
