# Supplementary material for: Improving Hox Protein Classification across the Major Model Organisms
Source: PLoS One. 2010 May 25;5(5):e10820. doi: 10.1371/journal.pone.0010820 (PMC2876039; doi:10.1371/journal.pone.0010820)
Supplement: Appendix S4 — CLANS network-clustering. Overview of the “network-clustering” approach as implemented in CLANS. Aim of this approach is to automatically identify groups of sequences with greater similarity to each other than to the rest. (0.24 MB PDF) [file pone.0010820.s004.pdf]

Outline of automated cluster detection via Network-clustering as implemented in CLANS

Aim of the automated clustering methods implemented in CLANS is to facilitate the selection of sequences and assigning them to groups. Displayed below is a typical cluster map.

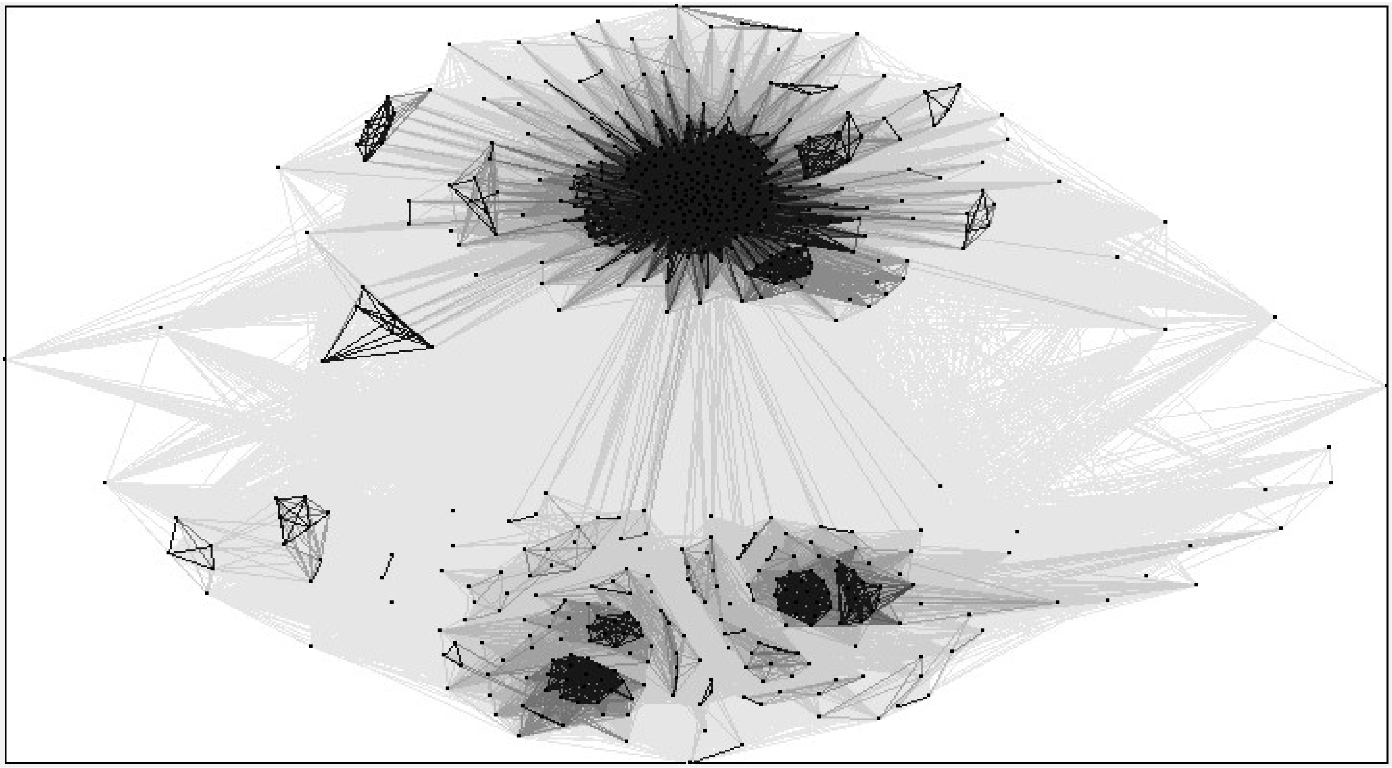

Network clustering detects 24 separate groups of sequences in this map.
Colors are used to highlight the different groups

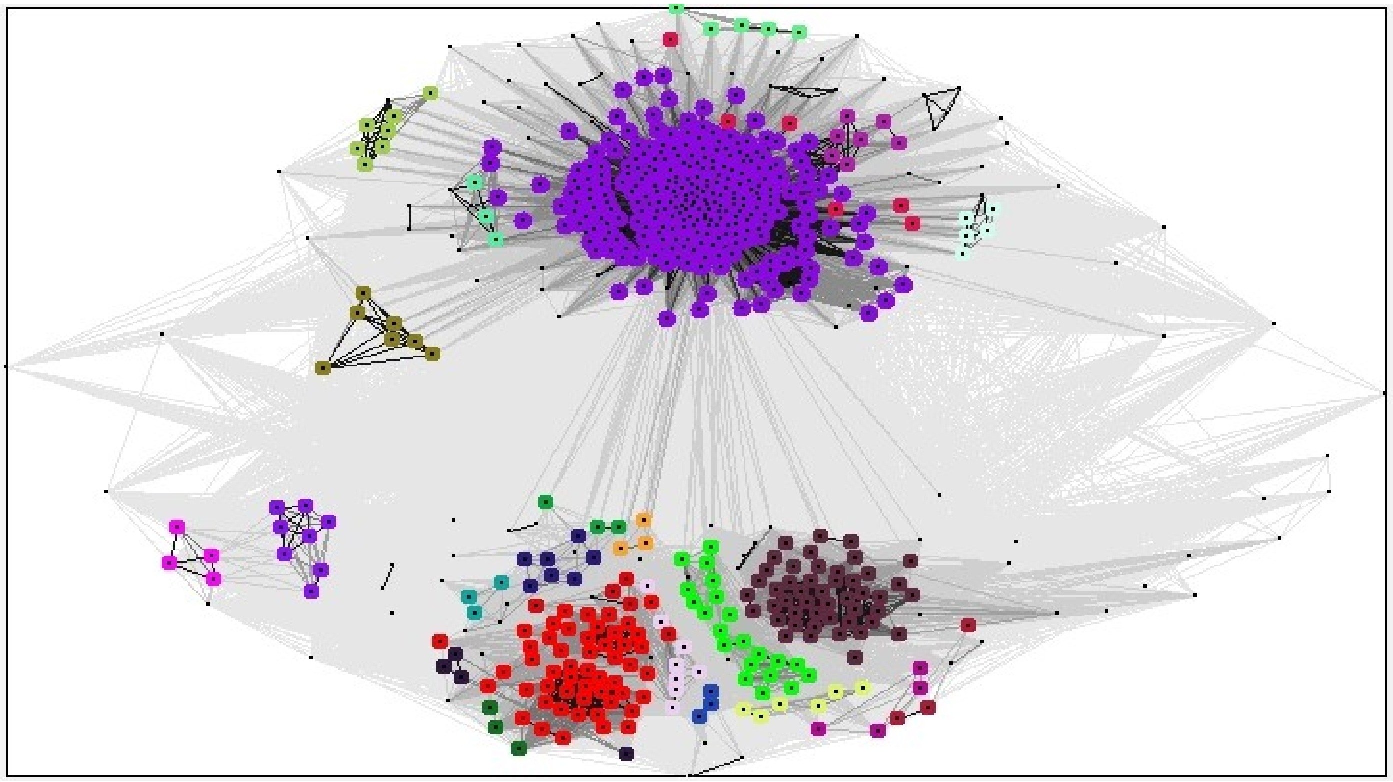

In the following, the clustering method is described using a simplified CLANS map (containing 10 sequences). The similarity values on which the map is based are, for simplicity's sake, either '0.9' (90% identity), '0.7' (70% identity) or '0' (below cutoff).

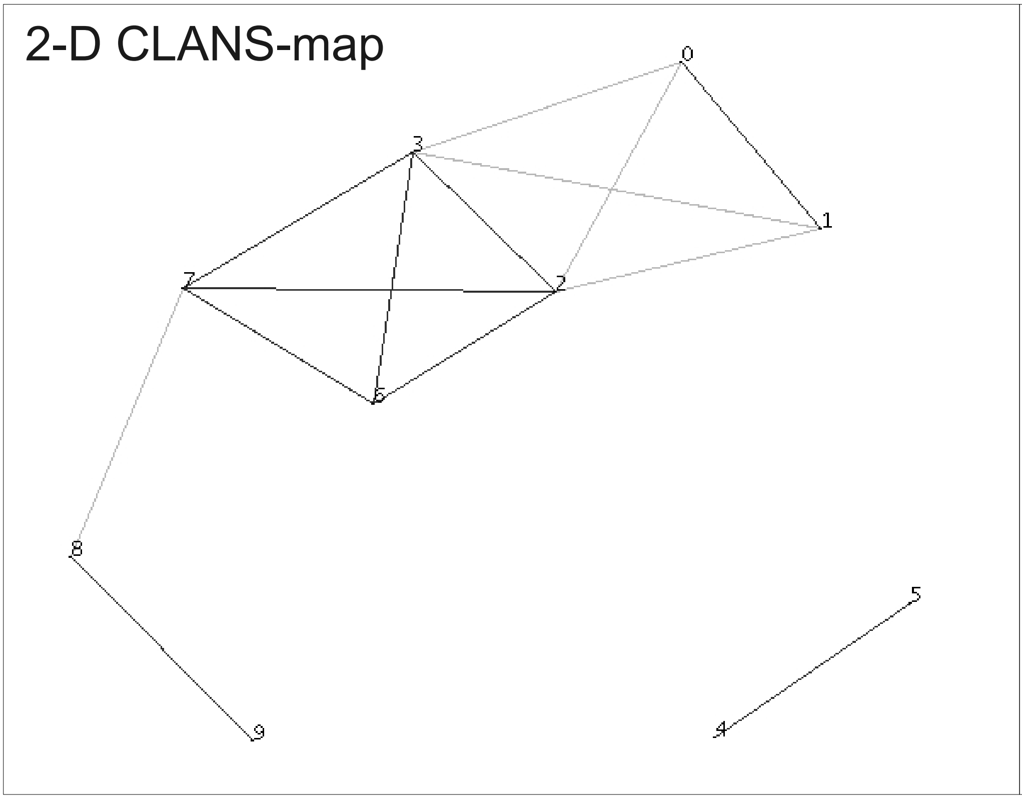

Map data:

Name1	Name2	Similarity
S0	S1	0.9
S0	S4	0.7
S0	S5	0.7
S1	S0	0.9
S1	S4	0.7
S1	S5	0.7
S2	S3	0.9
S3	S2	0.9
S4	S0	0.7
S4	S1	0.7
S4	S5	0.9
S4	S6	0.9
S4	S7	0.9
S5	S0	0.7
S5	S1	0.7
S5	S4	0.9
S5	S6	0.9
S5	S7	0.9
S6	S4	0.9
S6	S5	0.9
S6	S7	0.9
S7	S4	0.9
S7	S5	0.9
S7	S6	0.9
S7	S8	0.7
S8	S7	0.7
S8	S9	0.9
S9	S8	0.9

The 'Network-clustering' approach is implemented in CLANS as an iterative three-step process, but the data requires some preparation.

Preparation:

I) Each sequence in the CLANS map above (shown as a dots) is represented as a node/neuron in a single-layer, fully-connected neural network below. II) The connections between nodes are weighted according to the similarity values calculated for the sequence-pairs. In this example there are ten nodes (nodes 0-9) which are connected to each-other according to the above specified similarity values:'0.9' (black), '0.7' (grey) or '0' (no connection). III) Initially, each node is assumed to belong to a separate cluster: Node0 belonging to cluster-A, Node1 to cluster-B, ..., Node9 to cluster-J.

Preparation

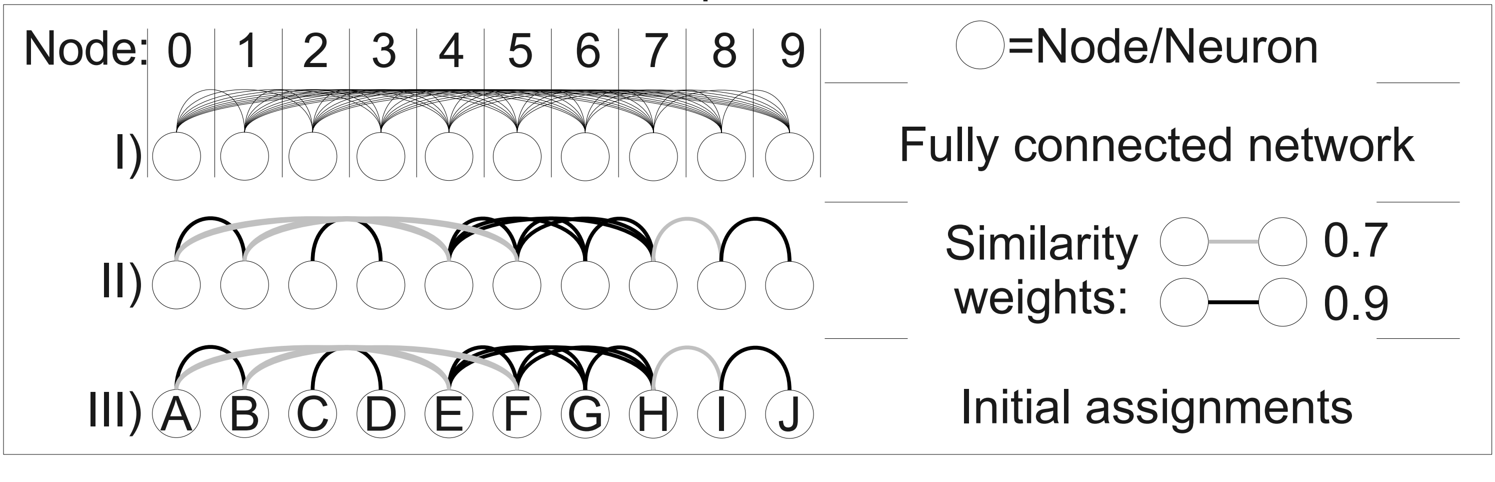

The subsequent clustering procedure consists of three steps, which are iteratively repeated until no further changes in cluster assignment of the nodes occurs.

Iteration 1

Step1 Emission:

Each node sends (emits) its current cluster assignment to every other node. This signal is weighted according to the weight of the connection between node-pair. Node0 would, for example, send a cluster-A assignment to Node1 with a weight of 0.9 and to Node4 and Node5 with a weight of 0.7 each.

Step2 Re-assignment:(IV)

Each node then adopts, i.e. is re-assigned, the cluster-assignment it received that had the highest weight. For example, the assignments received by Node0 consisted of: cluster-B with a weight of 0.9 (from Node1), cluster-E with a weight of 0.7 (from Node4), and cluster-F with a weight of 0.7 (from Node5). Node0 therefore adopts cluster-B as its new cluster-assignment. In cases where multiple incoming assignments have identical weights, see nodes 4-7, the cluster-identifier with the lowest value (cluster-'A' is lower than cluster-'B', 'B' lower than 'C', etc.) is given preference. In this case, Node4 adopts a cluster-F assignment and nodes 5-7 each adopt a cluster-E assignment.

Step3 Post-processing:(V)

In the previous step, many nodes swapped cluster-assignments. To avoid endless swapping back and forth, the post-processing step examines whether two nodes exchanged their assignments (i.e. they provided each-others highest-weight cluster-assignments) and, if so, both of them are assigned to the cluster with the lower-value identifier. Here: Node0 (formerly cluster-A, now cluster-B) and Node1 (formerly cluster-B, now cluster-A) are both assigned to cluster-A ('A' is lower than 'B'), Node2 (formerly C, now D) and Node3 (formerly D, now C) are both assigned to cluster-C, ('C'<'D') Node4 (formerly E, now F) and Node5 (formerly F, now E) both to cluster-E ('E'<'F') and Node8 (formerly I, now J) and Node9 (formerly J, now I) both to cluster-I ('I'<'J').

Iteration 1

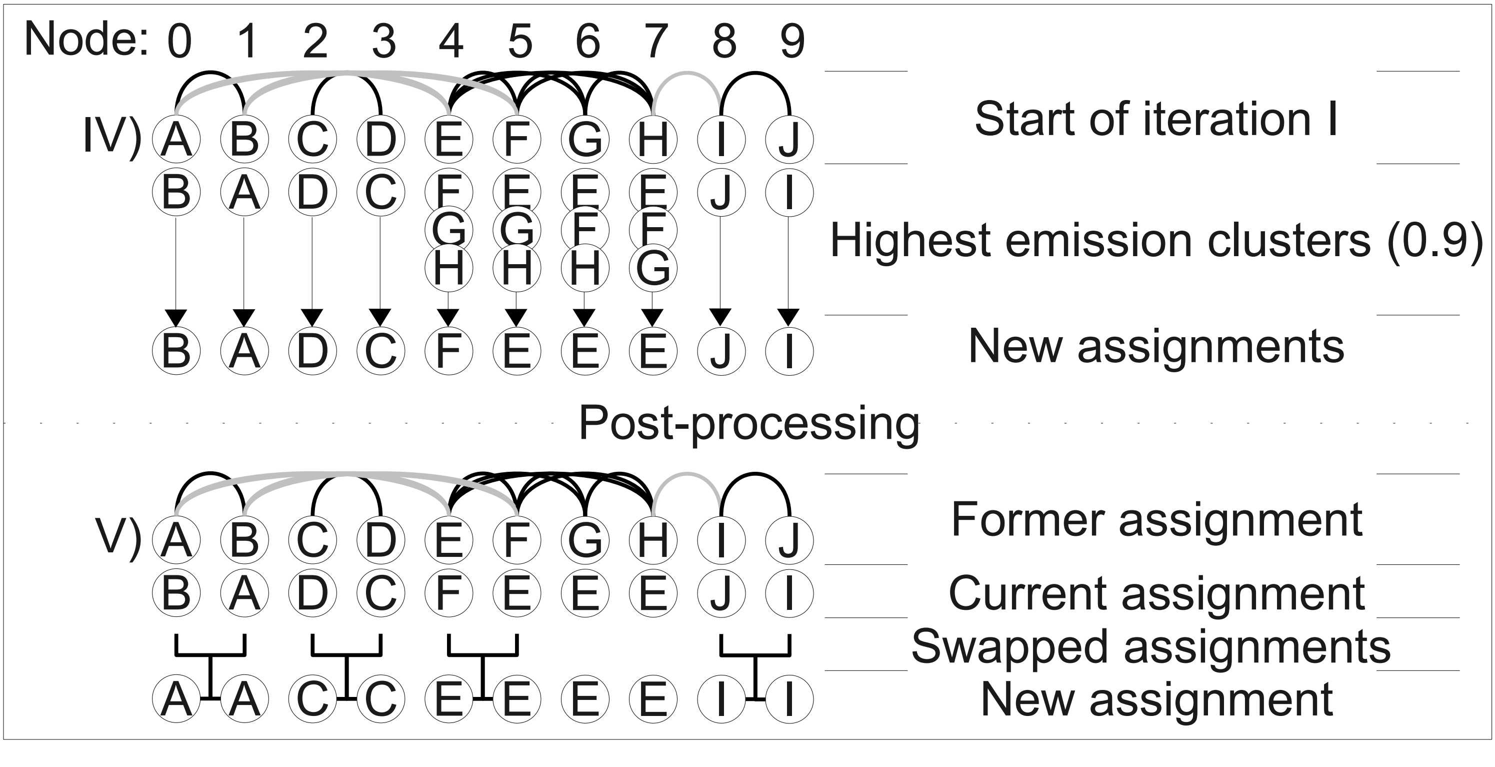

As some of the nodes changed cluster assignments, a further iteration is performed.

Iteration 2

VI) The cluster-assignments for nodes 0-9 at the beginning of the second iteration. Assignments received by Node1 are shown. Node0 sends a cluster-A assignment with a weight of 0.9 while nodes 4 and 5 both send cluster-E assignments each with a weight of 0.7 (2x 0.7=1.4). The cluster-E assignment weight is larger that the weight for cluster-A (1.4 > 0.9) and this causes Node1 to now adopt a cluster-E assignment (the identical scenario applies for Node0).

VII) The resulting post-emission cluster **assignments**: Node0 and Node1 have adopted cluster-E assignments, the assignments of the other nodes remain unchanged.

No **post-processing** (see iteration I, point 'V') is required as nodes 4 and 5 did not swap cluster-assignments with nodes 0 and 1.

Iteration 2

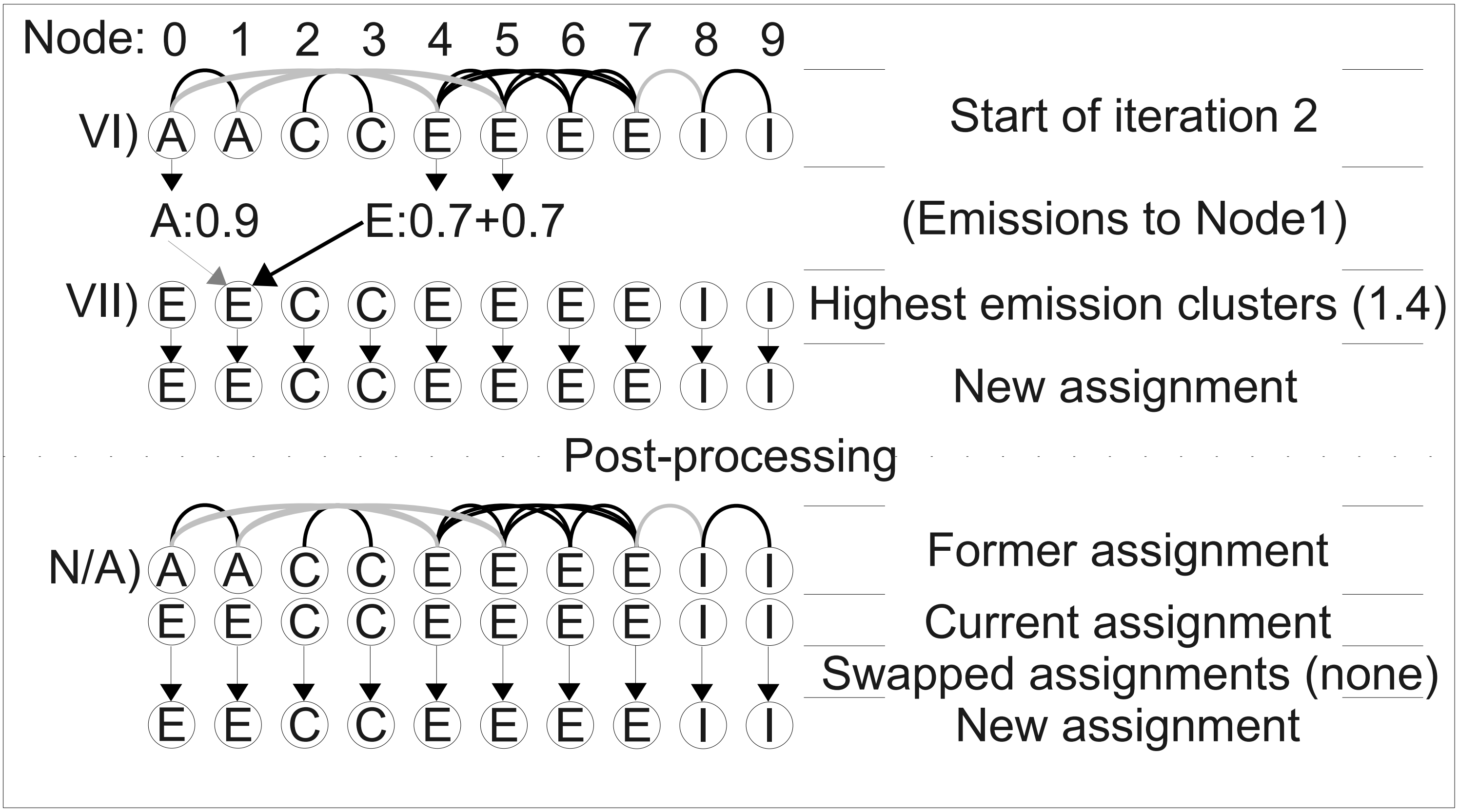

As some of the nodes changed cluster assignments, a further iteration is performed.

Iteration 3

(final iteration; no further changes in assignment occur)

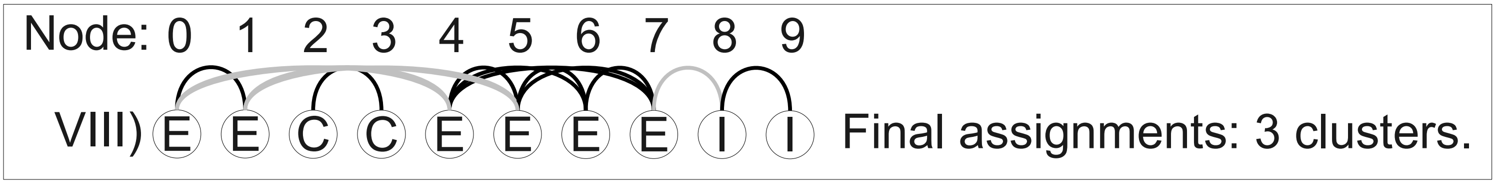

VIII) The ten nodes are assigned to three separate clusters:

Nodes: 0,1,4,5,6,7 to cluster-E.
Nodes: 2,3 to cluster-C.
Nodes: 8,9 to cluster-I.

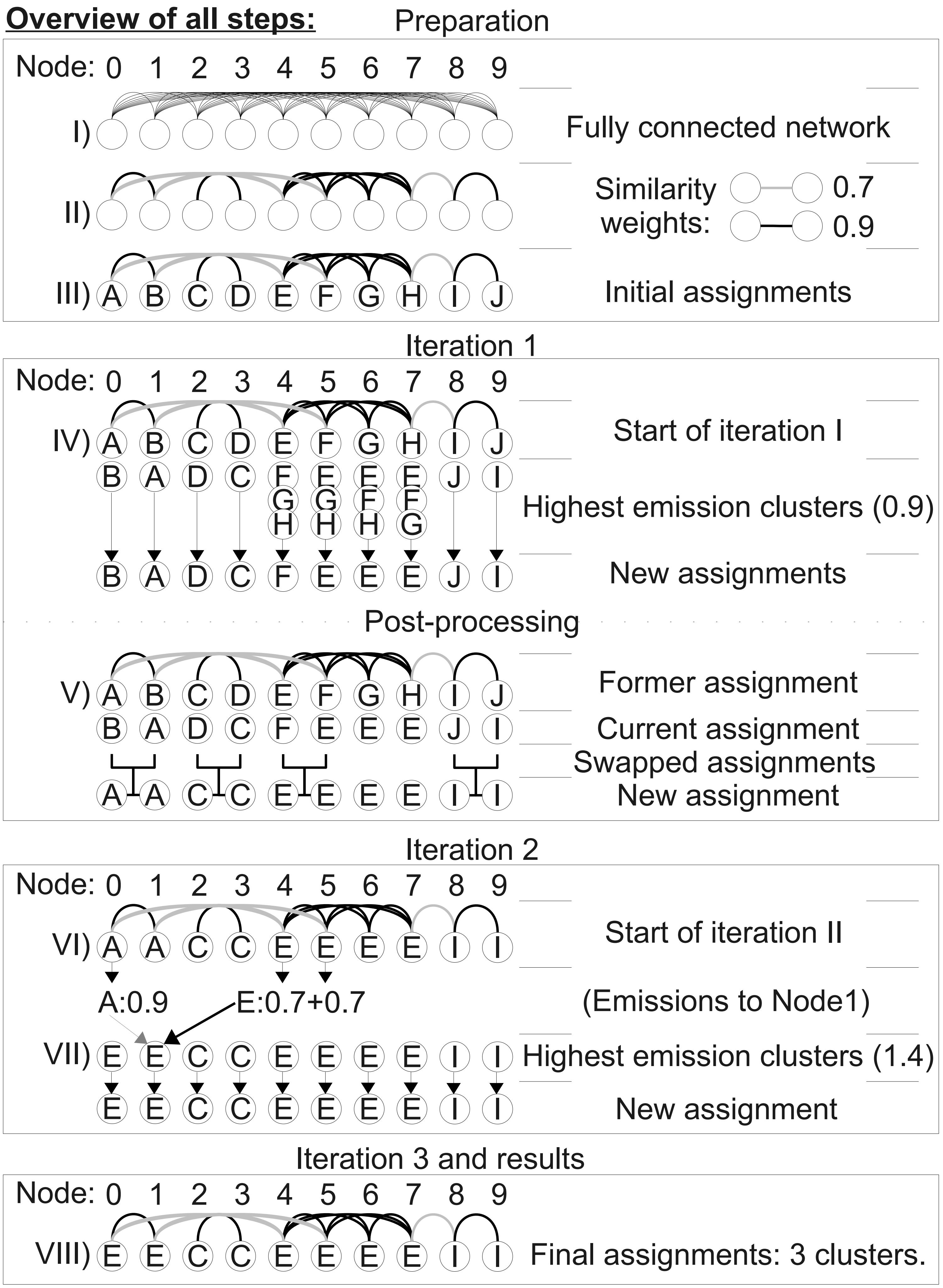
Overview of all steps:
Preparation
Node: 0 1 2 3 4 5 6 7 8 9
I)
Fully connected network
II)
Similarity weights:
0.7
0.9
III) A B C D E F G H I J
Initial assignments
Iteration 1
Node: 0 1 2 3 4 5 6 7 8 9
IV) A B C D E F G H I J
Start of iteration I
B A D C F E F F J I
G G F F
H H H G
Highest emission clusters (0.9)
B A D C F E E E J I
New assignments
Post-processing
V) A B C D E F G H I J
Former assignment
B A D C F E E E J I
Current assignment
Swapped assignments
A A C C E E E E I I
New assignment
Iteration 2
Node: 0 1 2 3 4 5 6 7 8 9
VI) A A C C E E E E I I
Start of iteration II
A:0.9
E:0.7+0.7
(Emissions to Node1)
VII) E E C C E E E E I I
Highest emission clusters (1.4)
E E C C E E E E I I
New assignment
Iteration 3 and results
Node: 0 1 2 3 4 5 6 7 8 9
VIII) E E C C E E E E I I
Final assignments: 3 clusters.
